# Planning and Evaluating Remote Consultation Services: A New Conceptual Framework Incorporating Complexity and Practical Ethics

**DOI:** 10.3389/fdgth.2021.726095

**Published:** 2021-08-13

**Authors:** Trisha Greenhalgh, Rebecca Rosen, Sara E. Shaw, Richard Byng, Stuart Faulkner, Teresa Finlay, Emily Grundy, Laiba Husain, Gemma Hughes, Claudia Leone, Lucy Moore, Chrysanthi Papoutsi, Catherine Pope, Sarah Rybczynska-Bunt, Alexander Rushforth, Joseph Wherton, Sietse Wieringa, Gary W. Wood

**Affiliations:** ^1^Nuffield Department of Primary Care Health Sciences, University of Oxford, Oxford, United Kingdom; ^2^Nuffield Trust, London, United Kingdom; ^3^Plymouth Institute of Health and Care Research, University of Plymouth, Plymouth, United Kingdom; ^4^Independent Research Consultant, Birmingham, United Kingdom

**Keywords:** remote consultations, video consultations, evaluation, telephone consultations, E-consultations, PERCS framework, complexity

## Abstract

Establishing and running remote consultation services is challenging politically (interest groups may gain or lose), organizationally (remote consulting requires implementation work and new roles and workflows), economically (costs and benefits are unevenly distributed across the system), technically (excellent care needs dependable links and high-quality audio and images), relationally (interpersonal interactions are altered), and clinically (patients are unique, some examinations require contact, and clinicians have deeply-held habits, dispositions and norms). Many of these challenges have an under-examined ethical dimension. In this paper, we present a novel framework, Planning and Evaluating Remote Consultation Services (PERCS), built from a literature review and ongoing research. PERCS has 7 domains—the reason for consulting, the patient, the clinical relationship, the home and family, technologies, staff, the healthcare organization, and the wider system—and considers how these domains interact and evolve over time as a complex system. It focuses attention on the organization's digital maturity and digital inclusion efforts. We have found that both during and beyond the pandemic, policymakers envisaged an efficient, safe and accessible remote consultation service delivered through state-of-the art digital technologies and implemented via rational allocation criteria and quality standards. In contrast, our empirical data reveal that strategic decisions about establishing remote consultation services, allocation decisions for appointment type (phone, video, e-, face-to-face), and clinical decisions when consulting remotely are fraught with contradictions and tensions—for example, between demand management and patient choice—leading to both large- and small-scale ethical dilemmas for managers, support staff, and clinicians. These dilemmas cannot be resolved by standard operating procedures or algorithms. Rather, they must be managed by attending to here-and-now practicalities and emergent narratives, drawing on guiding principles applied with contextual judgement. We complement the PERCS framework with a set of principles for informing its application in practice, including education of professionals and patients.

## Context: the Shift to Remote Consultations in the UK

On 30th July 2020, it was announced that all healthcare consultations in the UK should henceforth be “remote by default,” not just during the pandemic but indefinitely (1). Remote services had been introduced in March 2020 to manage the spread of COVID-19 and reduce the burden on the National Health Service (NHS). Patients seeking an appointment with their general practitioner (GP) had to make contact by electronic form or telephone before getting a return call from a clinician (2). In secondary care, much routine outpatient activity was canceled or undertaken remotely (3).

In the UK as elsewhere, the expansion of phone, video, and e-consultations were part of a wider pandemic-driven shift to technology-mediated care (4). These changes included directing patients to approved websites for self-management, an expanded telephone and electronic advice service featuring a bespoke COVID-19 Clinical Advice Service (CCAS) to supplement NHS 999 (for emergency calls) and NHS111 (for urgent and out-of-hours care), expansion of electronic prescribing, and introduction of virtual wards for oximetry monitoring (3).

Primary care clinicians welcomed the infection control benefits, empty waiting rooms, and slackening of red tape that accompanied the initial shift to remote (5). But they also warned of an uncomfortable “brave new world” characterized by fewer consultations overall, loss of continuity of care, threats to the clinician-patient relationship, inequalities of access, and clinical risks (6–9). Lay media coverage of remote consultations mirrored this pattern, with an initial positive response followed by stories of inaccessibility, missed diagnoses and patients feeling “fobbed off” with phone calls (10, 11). Whereas, politicians and the press emphasized the transformative potential of new technologies, most remote consultations before and during the pandemic occurred by telephone (3, 12–15).

Policy talk about remote health services was typically technology-focused, depicting these as state-of-the-art, high-quality, efficient, and safe (16). Arguments against remote forms of care tended to be patient-focused, highlighting possible disbenefits such digital exclusion. The benefit-harm balance of remote care is fundamentally an ethical issue. Technology-focused arguments often imply a broadly utilitarian ethical standpoint—that an efficient, remote-by-default service will minimize (though not eliminate) suffering, maximize overall benefit to the population and free up clinician time to create further benefits. Patient-focused arguments are more deontological, focusing more on what good care for the individual patient means, especially the clinician's duty to provide care for every patient to the best of his or her professional ability. However, critiques of remote modes of consulting can also be defended using utilitarian arguments, since clinical care is complex, relationship-based, nuanced, and emergent. If the consultation is narrowly transactional and fails to capture these wider dimensions, it is suboptimal, leading to inefficiency and exacerbating unfairness.

To guide and theorize our research on remote consultation services, our team set out to develop a conceptual framework. We began with a generic framework which some of us had developed previously, called NASSS—non-adoption, abandonment, and challenges to scale-up, spread and sustainability of technology-supported services (17). The NASSS framework has been widely used across a range of settings for evaluating and explaining the fortunes of various kinds of health technology projects [see for example (18–20)] and draws on complexity theory (21). NASSS explores the dynamic interaction between multiple domains (the technology, the people and so on) in a complex system and how these domains and their interdependencies have evolved—and are likely to evolve further—over time. There are theoretical parallels with other complexity-informed implementation and evaluation frameworks including i-PARIHS (22), normalization process theory (23), and the consolidated framework for implementation research (24).

But whilst NASSS was our theoretical starting point, we quickly discovered that it did not fully explain all of our empirical data—especially findings around the clinical relationship and the ethics of allocating appointment type. By adapting NASSS to fit our emerging empirical findings and relevant literature, we sought to develop a framework which would guide both the prospective planning and real-time evaluation of remote consultation services. Importantly, both NASSS and the adaptation described here (PERCS) are *explanatory* frameworks to guide a holistic interpretation of a complex and evolving phenomenon. They do not offer predictive certainty and are not intended to be applied formulaically.

The remainder of this paper is structured as follows. In section Empirical Context: Mixed-Method Studies of Remote Care Before and During the Pandemic, we describe the methods, study designs, sampling frames and datasets from our studies on remote consultation services in the UK. We highlight the elements of those datasets—especially qualitative research with staff and patients and an online Delphi study—which directly informed the theoretical work presented in this paper. In section Findings, we present and explain the PERCS framework and a set of guiding principles to inform its application. In section Discussion we contextualize our findings in a wider literature review and discuss implications for policy, practice, education and research.

## Empirical Context: Mixed-Method Studies of Remote Care Before and During the Pandemic

### Overview and Data Sources

Prior to the pandemic, we studied the organizational challenges associated with roll-out of video consultations across multiple clinical directorates in the UK's largest acute hospital trust (25–27), including sub-studies on physical examination by video (28, 29). We also undertook contract research for the Scottish Government to evaluate the national roll-out of video consultations—an initiative that was driven partly by the policy goal of reducing carbon footprint and travel costs from remote settings (30). Others in our team have studied help-seeking behavior in urgent care settings, including NHS 999 and NHS111 (31, 32). Insights from these studies informed our theoretical work.

Since the pandemic began, we have been involved in three separately-funded but theoretically related case studies. Details of ethics approvals are given at the end of the paper, and full empirical reports of these studies are in preparation for publication elsewhere. All studies were of mixed-methods design but predominantly qualitative, using interviews, ethnography, and documentary analysis to generate and follow an emerging story of change, using quantitative data to illustrate and enrich the story.

First, we were funded by the Scottish Government (June–October 2020) to extend our evaluation of the video consultation service (branded “Near Me”) to cover the early months of the pandemic to August 2021 (33). This study covered both primary and secondary care. It included 60 h of ethnographic observation; 223 interviews with healthcare staff, patients, and national-level stakeholders (policymakers, professional leaders, industry); quantitative analysis of automated activity reports on over 69,000 consultations (including over 18,000 patient assessments of consultation quality); and analysis of policy documents and implementation plans.

Second, we were funded by the UK Research and Innovation COVID-19 Emergency Fund from June 2020 to November 2021 for a study called Remote by Default, which addressed remote care in general practice. This study involves interviews (over 100 to date) with healthcare staff, patients and national-level stakeholders, as well as following four locality case studies in south London, Oxfordshire, Devon, and south Wales. Especially relevant to the development of PERCS were four online focus groups involving 19 participants (clinicians, support staff, and patients), four facilitated cross-sector workshops (held via Zoom) which brought together ~160 national policymakers, clinicians, patients, and other stakeholders, and a four-round Delphi study (described in detail below) of ethical principles and decisions relating to remote consulting.

Third, we were funded by a medical charity from June 2020 to July 2021 to study the roll-out of video consultations across the UK. The Health Foundation Video Consulting (HFVC) study involved a quantitative survey of current practice (to over 800 NHS staff), qualitative follow-up interviews with a sample of 40 of these (repeated longitudinally with a sub-sample of 20 as the pandemic unfolded), interviews with 10 patients, and two group discussions involving 15 patient and public representatives. This study also included 7 locality case studies of video consulting services—four in secondary care (in London, Norfolk, Oxfordshire, and Cumbria) and three on group video clinics in primary care (in England, Scotland, and Wales).

In each of these studies, our research question addressed the individual-, organizational-, and system-level challenges to introducing remote consultation services at pace and scale and routinizing such services. We used an embedded virtual researcher-in-residence model: each case study had an assigned member of the research team who built relationships with key informants, developed an understanding of local issues and contingencies, and coordinated data collection and feedback. An external advisory group with a lay chair and patient representation met 4-monthly.

### Developing the PERCS Framework

In all the above studies, we undertook an initial phase of data management and familiarization. Interviews, focus groups, ethnographic field notes, and workshop write-ups were transcribed, de-identified, entered onto NVivo software (or, in one sub-study, an Excel spreadsheet for pandemic-related practical reasons) and broadly coded (for example under headings such as “staff attitudes” or “technical infrastructure”). Quantitative data (e.g., waiting times, uptake rates and trends, survey responses) were analyzed using descriptive statistics. For each case study, narrative synthesis was used to pull together an initial familiarization document based on the first ~3 months' data; this document was refined iteratively as data collection progressed.

Researchers discussed their ongoing findings in 2-weekly team meetings. Initially we used NASSS (described above) to organize and theorize emerging findings, but when this proved inadequate for explaining some of our key findings, we began to adapt it into the PERCS framework. Particularly germane to this revision of our previous theoretical work were the transcripts and field notes from focus groups and workshops involving clinicians, support staff, and patients who discussed the challenges of remote consulting in the real world.

Across our in-pandemic datasets, a number of themes were evident which had received little or no emphasis in pre-pandemic research on remote consultations (see Discussion for summary of that literature). For example:

- *Access to care*, especially patients' difficulty getting the kind of appointment they wished and conflict with support staff associated with this;
- *Remote clinical assessment* of patients, especially those with a complex clinical picture such as evolving symptoms, co-morbidities, learning difficulties, cognitive impairment or vision or hearing difficulties;
- *Clinical risk management and safety-netting*, especially in acutely unwell patients (e.g., deciding which patients with suspected COVID-19 to see in person or send to hospital);
- *Continuity of care*, especially how to create coherence over time when different consultation modalities and more than one clinician were involved;
- *The patient's home and family context*, notably concerns about privacy and safety (e.g., whether patients are safe from eavesdropping or coercion);
- *Digital exclusion*, including the impact of poor-quality technologies, low digital literacy and material aspects of the home environment (and, even more so, being homeless);
- *Organizational efficiency* such as the nature of, and reasons for, “double-handling” (e.g., when patients were transferred from e-consultation to phone or from phone to video or face-to-face);
- *Staff wellbeing*, especially stress and loss of motivation from lack of face-to-face contact and a perception that consultations have become more transactional;
- *Technical issues*, including technical infrastructure; interoperability (especially interfacing with the electronic patient record) and in patients' homes (e.g., linking with multiple devices), and the wasted time and stress caused by unreliable internet access and technologies;
- *Sociotechnical issues* such as use of workarounds—for example, patients strategically downplaying symptoms on e-consultations to avoid an algorithm-driven diversion to a call handling service;
- *New forms of clinician-patient interaction*, such as the growing importance of asynchronous messaging between patients and clinicians (in the form of SMS, e-consultations, and emailing); and the unfamiliarity and strangeness of communication via video, including some people's dislike of seeing their own face or body on video and a sense of inappropriate intimacy (e.g., a doctor's discomfort at seeing a patient part-naked in a bedroom rather than on an examination couch);
- *Knock-on effects on vulnerable groups*, for example the impact on people with drug dependency (e.g., reduced in-person pick-ups for controlled drugs has resulted in fewer opportunities for direct clinician-patient engagement);
- *New opportunities for inter-organizational collaboration* e.g., for clinicians to engage in multiagency work to prepare and plan patient care; and
- *The national regulation and procurement context*, which shaped not only what technology might be available and affordable in any organization, but also who could use it and for what.

These themes were interdependent, illustrating the “complex system” aspect of the phenomenon we were researching. They informed development of draft PERCS framework as we used it to manage and synthesize data both within locality case studies and in cross-case analysis. We discussed this emerging work in workshops with our professional and lay advisory group members. Further refinements were made iteratively as we progressed with our analysis.

Two prominent themes in our data were organizations' digital maturity in providing remote consultations and the need for proactive measures to improve digital inclusion. We drew out different qualitative dimensions of digital maturity from our data, and also constructed a semi-quantitative digital maturity scale, incorporating work by others (34–37). Given that previous attempts at introducing a comprehensive digital maturity scale for the UK NHS had been abandoned as unworkable (36, 38), we deliberately favored a short, pragmatic five-point scale over a detailed, exhaustive one. We also teased out qualitative dimensions of organizations' efforts at digital inclusion and included reference to these in the digital maturity scale. To be classed as fully digitally mature, an organization thus had to address digital inclusion as well as install and use advanced technology.

### Developing the Guiding Principles

Evidence from policy announcements and our elite policymaker interviews revealed a relatively confident vision of an efficient, safe and accessible remote consultation service delivered mostly through state-of-the art digital technologies and implemented via rational allocation criteria and quality standards. But our front-line interviews, ethnographic observation, surveys, and service usage statistics revealed that, as in pre-pandemic times, the telephone was the dominant technology used and that decision-making at multiple levels—system-level strategic decisions about setting up remote consultation services, administrative decisions to allocate particular kinds of appointment (phone, video, e-, face-to-face), and clinical decisions when consulting remotely—was fraught with inherent contradictions and tensions. There were tensions, for example, between quality and efficiency, between demand management and patient choice, and between the needs and preferences of some patients and those of other patients in the context of limited staffing and material constraints (e.g., availability of safe waiting areas which limited face-to-face appointments during the pandemic). These tensions led to both large- and small-scale ethical dilemmas for managers, support staff, and clinicians. Few of these dilemmas could be resolved by resort to standard operating procedures or algorithms.

The contradictions and tensions in our data reflected clinical practice more generally. As Hunter (39) has argued, clinical decisions are governed not by hard and fast rules but by shared rules of thumb or guiding principles which she calls *maxims*, some of which are contradictory (e.g., “ignore the anecdotal” but “listen to the patient's story”). Maxims, which tend to be passed on orally from more to less experienced practitioners, encapsulate shared understanding and wisdom; they suggest a potential way forward but are high-level enough to be flexibly applied. Maxims and other high-level guiding principles require an understanding of the *circumstances in which the rule should be applied* rather than formulaic replication. Through experience, reflection on practice and discussion with more experienced colleagues, clinicians learn *which* guiding principle to use—and hence, what is the right thing to do—in a particular situation.

The situational application of such guiding principles has an important time dimension. With time, one constellation of symptoms, signs, and contextual influences will evolve to a subtly different constellation, generating clues as to the nature of the illness and its likely regression or further progression. A raised temperature observed at a single time point may persist, settle or become a swinging pyrexia. Only when this longitudinal pattern emerges do the relevant maxims to guide next steps become salient. Whilst Hunter focused narrowly on clinical maxims, case-based ethical reasoning more broadly—whether, for example, to invoke child protection measures or ask a social prescriber to get involved in someone's life—operates along similar lines. We need to know the story, its context, and how it is unfolding over time.

At the time of our fieldwork, there were few established guiding principles for provision of remote services (exceptions include “see all young infants face-to-face promptly” and “don't provide end-of-life care remotely”). To develop some more, we focused on a subset of our data relating to ethical tensions. We were aware of the many different ways in which ethical dilemmas in healthcare may be theorized—including utilitarian, rights-based, fairness or justice, virtue ethics, and the common good (40), and took the view that each of these philosophical lenses might prove useful in different scenarios. We also sought to consider ethical issues at individual-, organizational-, and system-level.

To develop and refine some ethical principles to guide application of the PERCS framework, we used the Delphi method—a well-established semi-structured approach to working toward consensus among experts (41). Steps include defining a problem, selecting a panel of experts (including, in this case, both clinicians and service users), supplying a summary of evidence and outlining key uncertainties, collecting quantitative (numerical scores), and qualitative (free text) data on a set of statements, feeding scores and comments back to panel members and repeating until residual disagreement cannot be resolved. Advantages of this method include practicality (it can be done online, asynchronously, without specialist tools), anonymity (participants know the average group score but not individuals' scores), and iteration (through feedback, outliers are prompted to either defend their response to the group or change it) (42, 43).

To prepare background evidence for the Delphi panel, we began by examining the tensions in our empirical data through various ethical lenses (presented in Findings). In this way, TG and RR developed a “long list” of 60 draft principles. We organized these into 30 partially contradictory pairs (for example, “when consulting remotely, deal directly with the patient and ensure privacy” and “relatives or carers may provide technical, linguistic, or physical assistance in remote consultations”) and grouped them under four categories (e.g., practice organization, matching appointment type with patient needs).

The four-round Delphi study was conducted virtually and asynchronously. Participants were recruited from our field sites, our advisory group and their networks, and a social media invitation (Twitter). We recruited and obtained emailed consent from an initial sample of 69, though 19 of these did not attempt any items (mostly because they found the exercise too difficult). The characteristics of those who participated are shown in Table 1; to avoid appearing intrusive we did not ask those identifying as patients or carers for details of occupation or illnesses.

**Table 1:** Sample for Delphi study of underpinning principles.

**Round**	**Professionals**	**Patients or carers**	**Total attempting at least one item**
Round 1	27 doctors, 4 nurses (2 of whom added that they were nurse practitioners), 1 physician associate, 2 physiotherapists, 1 health policy researcher, 1 from patient advocacy organization (total 36)	14	50
Round 2	22 doctors, 3 nurse practitioners, 1 physician associate, 1 physio-therapist, 1 medicolegal consultant, 1 policy researcher (total 29)	11	40
Rounds 3 and 4	21 doctors, 1 nurse practitioner, 1 physician associate, 1 physio-therapist, 1 medicolegal consultant, 1 policy researcher (total 26)	11	37

For round 1, we provided participants with a summary of research on remote consultations, guidance from professional and regulatory bodies (e.g., Royal College of General Practitioners, General Medical Council) and instructions. We invited them to consider each pair of guiding principles on three dimensions—whether they belonged in the assigned category; whether to include this principle as worded or in an amended form (scored on a five-point scale from “definitely include” to “definitely exclude”); and suggestions for improving wording. Since the list was long, we split it into two parts, each sent to half the participants in round 1.

Participants were given 3 weeks to complete the survey and were sent two reminders. Responses were collected on the survey platform Survey Monkey (pandemic-related home-working precluded access to more specialized tools). The software generated a single document with the spread of quantitative scores and collated free-text comments. We analyzed qualitative data thematically and through discussion. An initial plan to analyse quantitative data statistically was abandoned when it became clear that presenting our draft principles in deliberately contradictory pairs had led to confusion and irritation (which we discuss briefly in the Findings section). We used people's free text suggestions to drop many principles (merging those that were near-duplicates), improve wording and introduce several new principles suggested by participants.

In round two, we abandoned the attempt to present the principles in contradictory pairs. We circulated a simple list of 25 principles and asked participants to repeat the scoring exercise on two dimensions—whether each item should be retained (quantitative, five-point scale) and whether its wording should be changed (qualitative, free text). We used descriptive statistics to chart the level of agreement with each principle, and thematic analysis to organize free-text comments. This process was repeated in a third round. In a short fourth round, a single item (the only one on which 80% agreement had not yet been reached) was circulated to confirm a minor rewording suggested by several participants.

## Findings

### The PERCS Framework for Planning and Evaluating Remote Consultation Services

**Figure 1:**
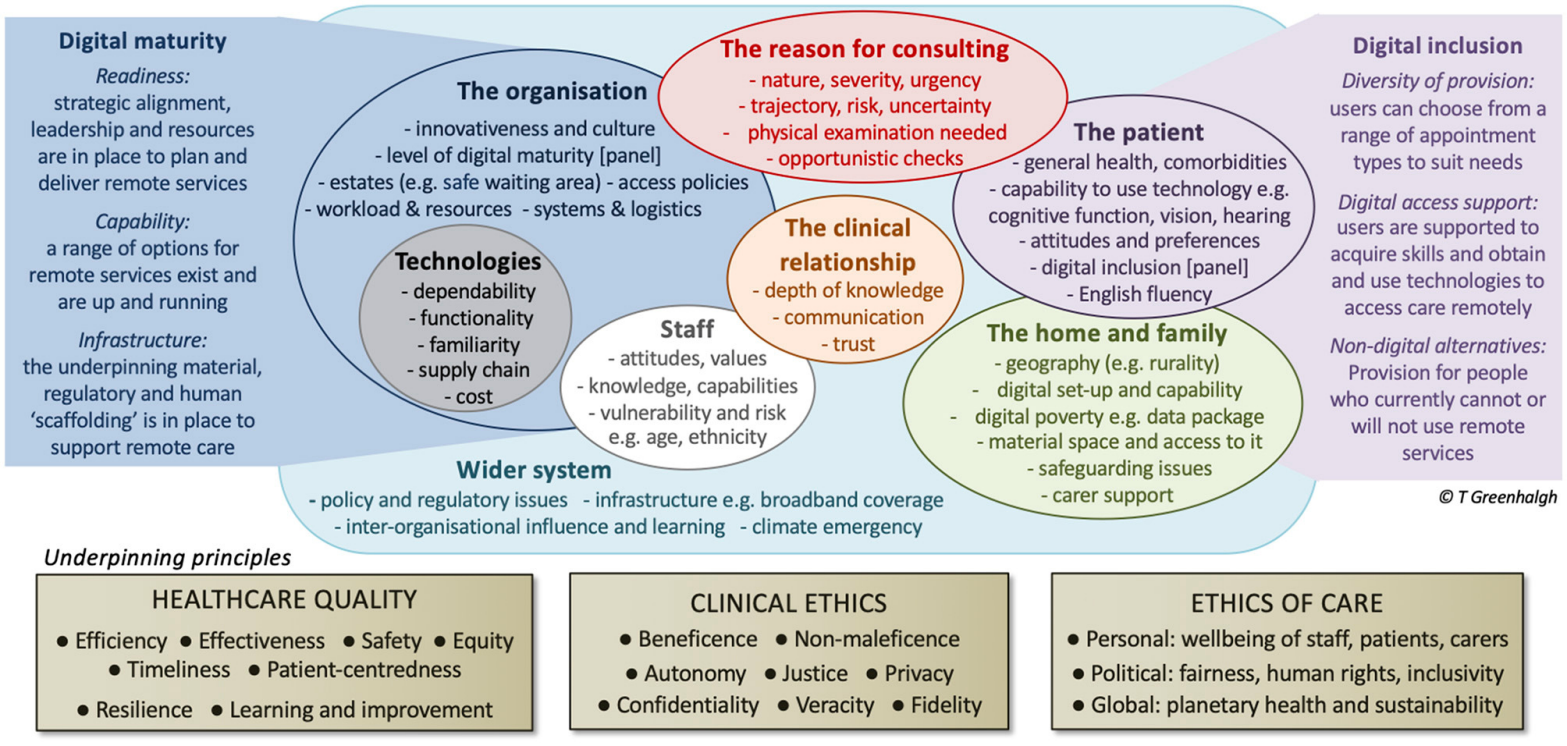
The PERCS (Planning and Evaluating Remote Consultation Services) framework and underpinning principles of healthcare quality and ethics.

The PERCS framework is shown in Figure 1. As noted above, it was adapted from the previously-published NASSS framework (17). We consider each PERCS domain in turn.

*The reason for consulting* considers not just the illness but why the patient wishes to be seen—or why the clinician wishes to see them—*now*. It draws attention to urgency and rate of progression, and to what the patient wants and expects (e.g., advice, treatment, referral). Relevant to this domain is the vast biomedical evidence base on the origin, progression and treatment of diseases and risk states, and sociological and psychological evidence on why people approach health services at particular points in their illness (44). Help-seeking for urgent care is a social process comprising illness work (to make sense of symptoms), moral work (to justify choice of service and help-seeking behavior) and navigation work (to access services) (31). Some reasons for consulting are clinician- or system-driven (e.g., invitations for screening or long-term condition monitoring). Clinical allocation criteria, perhaps built into algorithms, may suggest (though not determine) a type of appointment.

Several examples from our dataset illustrated the subtleties of the reason for consulting and why rigid algorithms or allocation criteria may prove too brittle to guide practice. Clinicians described over-riding such systems (for example when triaging e-consultations) because they had a gut feeling that a seemingly minor aspect of the history could indicate serious illness (45) or because they had safety-netting concerns that a particular patient should be checked more frequently than the recommended interval. They talked about the need to be alert to the possibility of the “doorknob phenomenon” —that people sometimes seek a consultation for one reason but bring in the real reason late in the encounter, hence withholding key information at the triage stage. The following quote illustrates the complex and subtle nature of gut-feeling reasoning (the doctor is highly reflexive and pulls together small fragments of the history and aspects of the patient's personality and past behavior, to justify their heightened concern):

“*there was somebody recently who was, you know, feeling very low, young man had kind of quite a long history of off and on of things … not always … minimizing it … very, you know, quite … I work in deprived areas quite … you know, quite, quite a high powered job, and in his 30s, and anyway I ended up thinking there's something more here, and so I brought him down to the surgery […] you know how you get a sense of stuff”* (GP, HFVC_BND)

*The patient* domain, which strongly influences the reason for consulting, includes the patient's (or parent's) attitudes toward illness in general and remote consulting in particular. It embraces the patient's identity, values, personality traits, and socio-cultural background—features a sociologist might call habitus (46)—, their health beliefs (44), health literacy and digital literacy, socio-demographic characteristics (47, 48) and their personal experience of illness or disability. All these factors may help explain why the patient seeks a particular mode of consultation—and also why a clinician or support staff member may decide that it is in the patient's best interests (or not) to be seen face-to-face, as the following quote illustrates:

“*I had a really interesting conversation with an 80-year-old with diverticular disease and he'd been phoning up every couple of weeks about his abdominal pain, which you routinely get with diverticular disease and there's no real cure and it's really just about managing his bowels. But nobody has sat down with him and drawn a section of the bowel and shown little pockets of diverticulae and how that's not gonna change and what he needs to do is to keep his bowels moving. So he doesn't understand what's going on for him and me trying to explain something over the phone like that is really hard to an 80-year-old who is losing every third or fourth word because his hearing is not brilliant anyway.”* (GP, RBD_FM3)

*The clinical relationship* domain considers the clinician-patient interaction, including what might be called non-transactional aspects of the consultation—for example, the role of the clinician as professional witness to suffering (49), or what Balint, writing in a more medically-dominated era, called “the doctor as the drug” (50). It addresses the level of trust and positive regard that can be developed and maintained in different types of consultation. It also embraces the clinician's—and perhaps also the administrative team's—knowledge of the patient and their illnesses and consulting patterns. This knowledge may have been built over many years (or, alternatively, may be recent and fragmented). One of the most frequent and consistent themes in our dataset was a sense from both clinicians and patients that the clinical relationship is necessarily built—at least initially—face-to-face. For example:

“*I think it's hard to build relationships over video and it's even harder over a phone call, OK? To really get to know somebody so that they trust you and you trust them and it's working. So, a lot of my phone calling is fine because it's people I've known for years because I've been in the same practice for eighteen and a half years and [distortion]. my patients.”* (GP, HFVC_IT)

*The home and family* domain incorporates considerations of how the material features, physical layout and symbolic spaces of the home influence key issues such as privacy and comfort when consulting remotely (51). Our datasets include examples of patients who had no home or whose homes were small, crowded, lacking privacy (notably houses of multiple occupation, but also many family homes under conditions of lockdown) or not connected to the Internet. One GP expressed concern that a patient was consulting from the lavatory because this was the only quiet and private space available. Another described connecting to a teenager's bedroom where a parent may have been present but off camera. This domain includes socio-cultural aspects of family life such as family structure, English fluency and the expectations placed on different members, and the extent to which family and neighbors contribute to the “lay consultations” that either promote or prevent a subsequent attempt to access formal health care (31, 52). It also includes wider social determinants of health, notably the level of socio-economic deprivation and the education and digital literacy of family members who may or may not be able to support the patient. More prosaically, consulting behavior patterns previously learnt in the context of the clinic must now be transposed to the home space, which may produce dissonance, as the following quote illustrates:

“*Well I had to like do. say to him, ‘Look, sit down, please sit down,' and then he. I just felt so daft and like, ‘Look, the doctor's going to tell you off if you don't sit down,' because of course [doctor] wasn't present. But that did kind of work. He kind of sat down and then he looked at her. But having him sat down and then like the others [siblings], you know, running around playing, is hard to prevent him from going off and playing.”* (Mother talking about trying to do video consultation with young child, HFVC_BV)

Aspects of the home and family domain—especially material spaces and privacy—may also be relevant to staff working from home during or beyond the pandemic. The only available space for one (female) GP to consult from was the kitchen table, necessitating complex negotiations with other family members about access to the food supply.

The domain of *technologies* includes aspects of design—such as aesthetic appeal, functionality, technical performance and ease of use—as well as dependability. Telephone, for example, has less functionality video but is more dependable and familiar. Some software products (e.g., accuRx) potentially allow the clinician to instantly convert a phone consultation to video in real time, thus avoiding double-handling, but products which lack this functionality require a new appointment.

The technology domain also incorporates supply chain and costs—including up-front investment, maintenance and repair, and the risks to the service if a key supplier pulls out of the market or raises prices (thereby embracing the “value proposition” domain from the NASSS framework). Costs of technologies are typically unevenly distributed across the healthcare system. Telephone advice services, underpinned by advanced computer decision support systems, can be costly to set up and maintain and require new work skills to operate (32); their introduction may generate supply-induced demand (53). During the pandemic, some locality-based clinical commissioning groups became tied into commercial contracts with video software providers whose products were approved at speed at the height of the crisis and offered “free” or low-cost for the first year.

The *staff* domain includes a quantitative workforce component (are there enough of the right kind of staff?) as well as a qualitative component relating to staff capabilities, confidence, attitudes, and well-being. Staff attitudes are grounded in professional norms and values including deeply-held concerns about quality and safety of care. Many staff—rightly or wrongly—still considered face-to-face consulting to be the “gold standard” professional norm:

“*They all miss face-to-face because that's what they were trained to do, they all work very, very closely with the children.”* (Children's community services manager, HFVC_IC)“

*And then also the barriers from, I think, healthcare professionals, so, 'How could I possibly have a difficult discussion over a video consult? How could I possibly discuss do not resuscitate [by video]? How could I break bad news when I can't touch my patients? How would I show empathy without touch? How can I be near and yet far? How could that be possible? You know, I need good eye contact, I need, you know, more non-verbals, I need to see the home, I need to see the person,' those sorts of thoughts maybe.”* (Palliative care consultant, HFVC_TQ)

Our findings affirmed published claims that remote consulting is cognitively burdensome (54), excludes the physical touch on which the therapeutic relationship has traditionally depended (55), threatens continuity of care (7), brings greater clinical uncertainty, and makes the personal care afforded by the extended, face-to-face “long consultation” more difficult (8, 56, 57). For example:

“*… it's maybe a slippery slope, you know, I'm trying my best in my consultations, I'm really focusing. I'm really tired from listening on the phone, because it is exhausting. I'm really tired from these Zoom meetings, because it's very different to have this sort of like overload of, you know, concentration and physically trying to do it, overload of information. And you feel like, you're failing, but actually, you're … but you can't do anything else to say, when you feel like you're not giving as good as services, as you know, somebody who was properly trained in all this, but you feel quite inadequate that, you know, but yet, you feel well, at least I'm doing something. But I need to be safe, and what are the unknown unknowns…?”* (Primary care nurse, RBD_IM)

Our interviews with less experienced clinicians confirmed published concerns from clinical trainees that the format of digital consultations has made it harder for them to observe and consult alongside more experienced practitioners, thereby accumulating the tacit knowledge that enables them to manage clinical risk confidently (58), as the following quote (reflecting on the collective learning that had happened pre-pandemic) illustrates:

“*…we were having clinical meetings in the surgery. We [had] a new doctor who joined us, who is young, who [had] just started, who [was] very hot on a weekly meeting that is mandated. And then she always [had] a sweet way of saying, Well, I'm the youngest here. I want to start with my worrying cases. case number one, case number two, what would you do here? What would you do there? And then, of course, we [would] all contribute.”* (GP, RBD_OS)

These complex effects of the remote modality on front-line staff added to the huge pandemic-related toll on their morale and well-being, which included the physical and emotional stress of clinical work, the personal risk of infection and its complications, and personal bereavements and other losses (59–61).

The *healthcare organization* domain incorporates the organization's general innovativeness and its readiness for a particular innovation (62). Innovative organizations tend to be large, competently led and with clear strategic vision, functionally differentiated, non-hierarchical and with adequate slack (people and resources that can be channeled into new projects to get them up and running). Readiness generally involves both top and middle-management support, absence of opponents, and a formal assessment of innovation-system fit (e.g., a business case), though such requirements were often relaxed during the pandemic. This domain also includes the complex question of how technologies become—or why they fail to become—taken up and maintained in organizations (23). Within the organization, the innovative technology or service model needs to be actively implemented and “normalized” or “routinized” (i.e., made business-as-usual), replacing one familiar and comfortable set of interactions with one that feels unfamiliar and awkward, as this quote illustrates:

“*I think we all miss. you didn't realize it at the time, but actually there's something very comforting about just having a [face-to-face] morning surgery booked and. we all moan about it but, you know, it's actually. you don't realize until it's gone actually how cocooned and in your zone you felt. And I guess it's how practices organize themselves.”* (GP, HFVC TU)

Normalization includes supporting staff to make sense of the new technology in the context of their work; engaging them to participate; coordinating their implementation efforts; and monitoring benefits and costs (23). Each of these phases can be time-consuming and exhausting, particularly when implementing technologies across multiple organizations (32). Small wonder that healthcare staff (both clinical and administrative) described themselves as “maxed out” and “knackered,” with little capacity for learning the new work routines needed for remote care.

Another aspect of successful organizational-level innovation is the need for staff members to champion and support the innovation. An example from our dataset is the administrative staff member who won an award for supporting the spread and scale-up of video consultations in community services. This digitally-literate individual grasped the vision for the new service, became an early champion for it and a super-user (someone to whom others felt they could go for help). They influenced fellow staff members to give it a try (including calling in favors), developed bespoke training for colleagues and patients, and pushed for measures to help embed the change (e.g., better digital platforms) with support from management. Slow adoption of remote services in some other organizations or departments could often be attributed to absence of such individuals: nobody opposed the innovation, but nobody enthused about it either.

The *wider system* domain incorporates the powerful phenomenon of inter-organizational influence and learning, in which early-adopting organizations pass on insights and resources to those coming on stream later, as well as attention to how policy context may support—or interfere with—organizational innovation (62). An example of cross-system influence at national level was how Scotland, which had been an early and successful adopter of video consulting across the country, supported the other UK jurisdictions (Wales, England, and Northern Ireland) with advice and re-usable resources (e.g., protocols, patient information). Learning from models of good practice elsewhere tended to be a positive experience, whereas public release of performance data—in which each organization or department is compared competitively with others (for example in the proportion of consultations undertaken remotely)—was viewed negatively by our participants.

An important aspect of the policy context for remote consultations during the pandemic was the use of emergency powers to slacken red tape in relation to approved suppliers and purchasing rules, allowing organizations to effectively obtain and use any system deemed locally appropriate and workable (63).

“*…essentially, we were given the word from the Trust that had come down from the region saying, 'Look, all is forgiven so to speak. For the time being, till we get a proper governance structure in place, just anything goes.'”* (Service manager, HFVC_QA)

Our interviewees depicted these changes positively—indeed, they considered that the changes would have been impossible or much delayed without them—but they also expressed concern about what would happen when organizations returned to business as usual, since the longer-term trade-offs between convenience and regulatory control (e.g., data privacy) are unclear. Profit-oriented providers offering only a limited range of remote services and targeted primarily at low-risk patients such as young professionals were part of the landscape before the pandemic, competing with local GP practices. Their success during the pandemic at a time of regulatory laissez-faire raises questions about quality, safety, and equity of care going forward (64).

The wider system domain also includes policies that affect remote services indirectly. An example is cross-government measures to address the climate emergency through a range of green policies. NHS England, for example, recently announced a vision for a “net zero [carbon] NHS,” including a major contribution of new modalities for delivering care (65). The evidence base for achieving greener health services by means of remote care is limited and contested, but one study suggests that a substantial reduction in carbon footprint could be made (66).

Another aspect of the wider system domain in PERCS is underpinning infrastructure. Even before the pandemic, there was a strong policy push in the UK to build and strengthen the enabling technical infrastructure for a digitally-supported NHS (67–70), resulting in some relatively state-of-the-art elements such as the Health and Social Care Network (referred to as “The Spine”), along with various local and regional legacy components (some of which posed challenges relating to coverage and interoperability). But as we found, not everywhere in UK has even basic broadband connection:

“*we're in rural Northern Ireland, there is still areas of the peninsula that haven't got Wi-Fi; they have got no sort of connection to things. Broadband speeds in many parts of Northern Ireland are one megabyte per second. So, just video consultation just physically doesn't work.”* (Service manager, HFVC_QA)

Infrastructure also includes human elements (e.g., organizational roles, routines, and relationships), shared understandings and historical path-dependencies (such as legacy technologies and commercial contracts), and the regulatory and professional standards noted above (27, 37). As Gkeredakis et al. (4) have pointed out, during the pandemic a sense of urgency, relaxing of regulations and policy investment in technological solutions produced many new technological components but there was not time to strengthen either technical or human infrastructure, yet successful embedding and use of these novel solutions will be “*contingent upon the openness, distributedness, recombinability, re-programmability, and accessibility of digital technologies”* (page 2).

The initial draft of the PERCS framework consisted of the seven domains listed above, along with a temporal domain in which the dynamic interaction between all the other domains is followed over time, using narrative as a summarizing and synthesizing tool. One England-based clinician, for example, reflected that whilst video services had been broadly successful during the pandemic, there was little enthusiasm from her colleagues to continue this mode, and she doubted the service would last beyond the pandemic. She planned to return to Scotland where there was a long-term strategy and vision to extend video-based clinical services.

We subsequently added two side panels to the PERCS framework to place special emphasis on *digital maturity* (of the organization) and *digital inclusion* (for the population it serves). Whilst these concepts are to some extent subsumed within the central domains, the terms are increasingly widely used in healthcare circles and are worth exploring in their own right.

The theme of digital inclusion recurred in our data. For example:

“*We have pockets of significant deprivation in [City name] and people just don't have the IT capability or the connectivity. We've had a lot of difficulty around connectivity, and I am not IT, so I'm not sure if that is largely down to the device that a patient or service user has, or whether it's down to the actual Internet or, you know, whatever the infrastructure is for. do you know what, I actually don't know the terminology but you know what I mean?”* (Hospital clinician, HFVC_EY)

“*…particularly with the with the outreach population, it's so easy to ignore them. You know, they are not jumping up and down and making a fuss and they can't jump up and down and make a fuss because they can't fill the forms in, because they've got to have internet access to fill the form in to complain. Yeah, it's really it's quite insidious. Really, how it, how it excludes people, and then it stops people complaining about it.”* (GP, RBD_CK50023)

Digital inclusion should be considered in relation to inequalities more generally. Tudor Hart's (71) inverse care law states that people most in need of health care are least likely to seek it or receive it; the law reflects two mutually-reinforcing phenomena—worse health in deprived localities and barriers to accessing healthcare in those same localities (47, 48, 72, 73). Patients who already suffer the multiple jeopardy of poverty, low health literacy, poor housing, weak social networks, psychological stress (e.g., from fear of crime) and—for some—language and cultural discordance now face an additional problem of digital inequalities, defined as differential access to healthcare depending on digital access, digital literacy or both (74). These barriers are part of a wider digital shift whereby many aspects of life—insurance, banking, local government, education, travel, and holidays, and many more—are increasingly presented to the citizen, client or customer as “digital first,” thereby excluding (partially or wholly) those unable to access them in this way (75).

The digital divide needs to be studied at a granular level, not merely in terms of the presence or absence of Internet access (76) but also in terms of how much bandwidth, data, IT literacy and skills, and power (e.g., over who in the household has use of the computer or smartphone) people have (77, 78). Digital inclusion is high on the policy agenda in the UK and elsewhere (79, 80). Some groups (e.g., the neuro-atypical and some people with mental health conditions) are said to feel more comfortable with remote consultations than face-to-face, though anecdotal evidence outweighs rigorous research on this topic (81).

Drawing on previous research and policy work (76, 78, 80, 82–84) as well as our own empirical data, we conceptualized digital inclusion as requiring three kinds of measures. First, diversity of provision, to ensure that patients and carers are able to select from a wide range of options to suit their particular access needs and preferences; such options would ideally be co-designed with patients and carers. Second, digital access support—either directly from the organization or indirectly by signposting to third-party or community providers (libraries, community networks)—providing access to the equipment needed for remote services; help to acquire and use the skills to engage with digital health care and support to create coherence between multiple providers offering digital services in different ways. This might include upskilling individuals but also, where necessary, major infrastructural projects to improve broadband connection to remote localities. Third, provision of non-digital alternatives for people who are unable or currently unwilling to access care remotely.

Examples of digital inclusion efforts in our dataset included flexibility to accommodate patient preferences (some teenagers, for example, preferred to consult with the video switched off; some parents preferred to video their child doing a task and send the video to the therapist rather than the child perform live to camera), signposting to local digital literacy programmes, and making it clear to patients that face-to-face appointments were available on request. Co-design with digitally excluded groups is recommended by advocacy organizations (82). However, our dataset included numerous examples of capacity constraints which limited organizations' abilities to undertake co-design work, provide a high degree of flexibility or provide sufficient face-to-face slots to accommodate all requests.

Healthcare organizations participating in our studies were at different levels of digital maturity (for which a dictionary definition is “a measure of an organization's ability to create value [financial and non-financial] through digital”), as the following contrasting quote illustrates:

“*When the COVID hit, obviously we had access to video consulting and we got on with it, but it very quickly petered out. So, we went to total telephone triage; we did some video consults. We got pictures sent to us via email and things like that. The functionality of our platforms … was generally quite poor; had a lot of problems connecting. So yeh, we did it for a bit, but I would say that we never exceeded probably ten percent of what we were doing actually using video. And now I don't use video at all. Only one of my partners is continuing to use it now and again.”* (GP, HFVC_YN)

Our digital maturity scale is shown in Table 2. It has three linked elements. First, *readiness*—the extent to which the organization has the strategic alignment, leadership and resources needed to plan and deliver an appropriate range of remote services, including measures to address digital inequalities. Second, *capability*—the extent to which such services, including digital inclusion measures, are already technically present and up and running. Third, organizational *infrastructure*—the extent to which the underpinning material, regulatory, and human resource frameworks are in place to support further development of remote services.

**Table 2:** Digital maturity scale for healthcare organizations in relation to remote services.

**Organizational descriptor**	**How the organization currently uses traditional technology (e.g., phone, online access) and new technology (e.g., video, telehealth apps) to support remote consultations**
Level 1: Traditional (reactive)	Limited leadership or vision for remote services (there may be a strategic decision and rationale to resist these). Phone is used for triage and call-backs e.g., for demand management and as a response to the pandemic. Patient online access is mostly disabled. Video and telehealth are rarely if ever used and may be actively discouraged. Key infrastructure may not be in place. Digital inequalities either not addressed or addressed by focusing on face-to-face services.
Level 2: Traditional with lone innovator (*ad hoc*, demonstration)	Within a traditional organization or department, one staff member is enthusiastic about remote care, s/he attempts to use novel technologies and engage others in doing so, but has not yet succeeded in getting others to share the vision, influencing practice strategy or changing practice routines or policies. Infrastructure may be inadequate. Digital inclusion not yet a priority issue.
Level 3: Digitally curious (experimenting)	The organization or department has a vision and plans for providing remote care. Traditional and new technologies are used creatively, and adjusted iteratively, to try to improve an aspect of care within the practice. These creative efforts may include measures to overcome digital inequalities. Focus is on technical details and feasibility (i.e., making something work). Infrastructure is adequate but may have limitations.
Level 4: Digitally embedded (learning and improving)	Both traditional and new technologies are used creatively and strategically, and benefits and disbenefits are evaluated, with the aim of improving remote care in all relevant areas across the organization, including efforts to meet the needs of digitally excluded groups. Digital capability is high (i.e., many services are successfully delivered remotely). Focus is on quality improvement and organizational learning. Work practices and routines are continuously adapted. Technical infrastructure is good as a result of strategic investment.
Level 5: System-oriented (extending and spreading)	Strategy and vision for remote services are strong and extend beyond the organization itself. Reducing digital inequalities is one aspect of a wider vision for an effective, efficient, equitable remote service. Digital capability is high. Staff are actively involved in developing and evaluating remote services beyond the practice—e.g., through inter-organizational benchmarking, quality improvement collaboratives, locality-wide planning, research, national guidelines.

### Findings on Practical Ethics of Remote Consultations

Evident in our interviews with clinicians were the four widely-cited principles of practical medical ethics—beneficence (act in the patient's best interests), non-maleficence (do no harm), autonomy (respect the patient's right to choice and self-determination), justice (treat people fairly, especially when allocating scarce resources). These principles were originally proposed by Beauchamp (who leant toward a consequentialist or utilitarian position, focusing on outcomes of decisions for the patient and others) and Childress (a deontologist focusing more on the clinician's duties) (85), and extended to consider scope of application by Gillon (86). Beauchamp and Childress (87) later added four further principles based on behavioral norms—veracity (tell the truth), privacy (ensure no intruders or eavesdroppers), confidentiality, and fidelity (avoid conflicts of interest such as the potential for personal profit). All these principles featured prominently—often as professional norms assumed to be self-evident—in our empirical data. They are reflected in guidance such as the General Medical Council's *Duties of a Doctor* (88), to which some clinical interviewees referred.

Additional ethical tensions related to staff well-being and redistribution of the work of caring (often shifting to the patient or lay carer). These themes resonated with writing by feminist philosophers (89–92) on the ethics of care, including the hidden work and emotional labor of low-status workers such as receptionists and unpaid carers. Held's taxonomy (91) divides the ethics of care into personal (focusing on an individual's commitment and accountability to the person they are caring for), political (focusing on various kinds of inequality in caring—such as fairness, human rights, and inclusivity) and global (caring for the planet and its sustainability—and hence caring for future generations).

A related concept is what May et al. (93) have called burden of treatment—the additional burden placed on the patient when they are asked to “self-manage” —with the sickest and most vulnerable carrying the greatest burden. Burden of treatment also includes the effort needed from the patient to access services (31, 93), which technologies (especially e-consultations) could potentially alleviate. Pols (92) has developed what she calls an empirical ethics of [technology-supported] care which considers technologies not as inanimate tools which make the clinical interaction more or less efficient (and as “cold” artifacts that contrast with “warm” human care) but as relational actors which, if creatively selected and “tinkered with” to fit specific situations, can *enhance and support* the personal ethical commitments and warm care relations between people.

Finally, our empirical data raised organizational- and system-level ethical issues, many of which could be mapped either to the US Institute of Medicine's (94) six dimensions of quality—safety, efficiency, patient-centeredness, timeliness, effectiveness, and equity—or to more recent work on organizational and system resilience—for which redundancy may be needed to help weather stress—and sustainability (95, 96). While these are quality principles rather than ethical principles, they draw implicitly on many of the ethical principles described above and have become widely used by those planning, developing, and evaluating services. These various ethical and quality frameworks were added as underpinning pillars to the PERCS diagram.

Of the 50 participants who completed round 1 of the Delphi survey, many put in free text comments that the paired principles were “contradictory” and “confusing.” This had been our intention, but it was clear that whilst clinicians work comfortably with contradictory principles in their day to day work, they experienced strong cognitive dissonance when asked to confront them in a desktop exercise without a real, practical situation in mind.

All but one of the 25 revised principles in round 2 were supported in principle by 80% or more respondents, but many had suggestions for changes in wording (e.g., be less paternalistic, reduce jargon, split a maxim into two). All but one of the 26 revised principles in round 3 were supported by 80% or more participants with only minor suggestions for further revisions to wording; the final item was supported by 71%, with several respondents all suggesting omission of a particular phrase. After re-circulating this one item in a brief fourth round, there was high agreement on all 26 principles, which are listed in Table 3.

**Table 3:** Guiding principles to inform application of the PERCS framework.

**SECTION A: PRINCIPLES UNDERPINNING OUR REMOTE SERVICE**
1. Infection Control (97% agreement)
We take all reasonable measures to ensure safety when there is an infection risk, including providing a range of ways for patients and staff to consult remotely.
2. A Fair Appointment System (97% agreement)
We have systems in place to allocate appointments fairly, prioritizing the most urgent. We take account of the fact that some patients are unable to use some or all types of remote consultation.
3. Informing Patients (97% agreement)
We provide information for patients on the different kinds of appointment available and the circumstances in which these may be appropriate. When offering appointments, we can explain why we think a particular type is suitable.
4. High Clinical Standards (91% agreement)
We are committed to providing the highest quality of clinical care for all patients, whatever the type of consultation.
5. Balancing Benefits and Risks (94% agreement)
We recognize that remote appointments have a different balance of benefits and risks—for example, greater convenience but less opportunity for physical examination, safe disclosure of sensitive information or raising potentially serious symptoms.
6. Technical Security And Usability (89% agreement)
The technologies we use for remote consulting meet high standards of data privacy and security while also being easy to use. We may support occasional use of less secure technologies that are more familiar to patients where benefits of doing so outweigh risks and patients accept these risks.
7. Patient Centredness (94% agreement)
Subject to capacity, we endeavor to offer all patients an appointment type which is timely, acceptable to them and addresses their needs.
8. Staff Wellbeing (88% agreement)
We manage appointments so as to take account of staff workload and wellbeing. As far as possible without compromising patient care, we allow clinical staff to choose their preferred mode of consulting (e.g., taking account of their own clinical risk and stage of training).
9. Environmental Responsibility (94% agreement)
Our policies on remote consultations reflect our commitment to reduce unnecessary travel and contribute to a greener, more sustainable future.
**SECTION B: GUIDANCE FOR STAFF—BEFORE THE CONSULTATION**
10. Deciding on Appointment Type (92% agreement)
Where possible and appropriate, we offer patients their preferred type of appointment. When allocating appointment type, we take account of the reason for the request and have processes in place to identify contextual factors, including but not limited to
- Whether the patient is known to the practice team - Whether there is access to their full medical record - Communication needs e.g., visual or hearing impairment, literacy issues, difficulty understanding, need for interpreter - Privacy or safeguarding concerns - Infection risk
11. Reducing Double-Handling (89% agreement)
We have measures in place, including effective triage, to ensure that patients are efficiently directed to an appropriate consultation type. Some phone or e-consultations will need to be followed up with a different type (e.g., face-to-face).
12. Making Complex Judgements (93% agreement)
Since each appointment request is unique we encourage staff to use their judgement and discuss decisions with patients and with a senior colleague where appropriate.
13. Mitigating Digital Exclusion (97% agreement)
When allocating appointment type, we try to take account of - The patient's access to private space for a remote consultation - The patient's digital set-up - Their capability and willingness to use different kinds of technology
If patients are unable to manage a particular type of remote appointment, we offer a suitable alternative.
14. Supporting Continuity of Care (94% agreement)
Subject to resources, we aim to support
- Continuity of relationship (with own clinician) - Continuity of information (of the patient record) and - Continuity of multidisciplinary care (across a team)
15. Embracing Uncertainty When Allocating Appointments (87% agreement)
We recognize the uncertainty associated with allocating remote appointments. For example, we are alert to - Patients who are concerned about seemingly minor or non-urgent complaints - Patients who are keen to have a face-to-face appointment but do not wish to give a reason - Patients whose condition has not improved following remote consultation(s)
16. Addressing Complex Needs (93% agreement)
We try to ascertain and address the particular access needs of patients who may be vulnerable (due—for example— to multiple medical conditions, advanced frailty, learning difficulties or cognitive impairment) in a compassionate and flexible way.
17. Safeguarding (100% agreement)
We are sensitive to the possibility that remote consultations may be compromised through interference or coercion. If there are such concerns, we offer a face-to-face appointment.
**SECTION C: GUIDANCE FOR STAFF—DURING AND AFTER THE CONSULTATION**
18. Supporting High-Quality Interaction (94% agreement)
When consulting remotely, we allow time for both parties to optimize the connection, deal with technical glitches and check understanding. We recognize that it may be harder to convey empathy and build therapeutic rapport in remote consultations.
19. Balancing Patient Autonomy with Support from Carers and Friends (100% agreement)
When consulting remotely, we deal directly with the patient if possible and respect their privacy. Subject to consent, and mindful of safeguarding issues, we welcome input from relatives or carers to help with the technology, communication or a remote physical examination.
20. Embracing Uncertainty During the Remote Consultation (94% agreement)
We recognize the uncertainty associated with assessing and managing patients in remote consultations. For example, we are alert to - Poor audio or video quality - Absent or limited visual cues - Patient or practitioner stress - Limited scope for examining the patient - Possible presence of a third party off camera
21. Remote Physical Examinations (100% agreement)
When considering whether to examine the patient remotely (e.g., by video or by asking them to take measurements), we take account of - The level of urgency - The patient's comfort and confidence - Their ability to assist (e.g., by placing a camera) - Whether relatives are—with the patient's consent—able and willing to help
We invite patients to attend in person if an adequate physical examination cannot be done remotely.
22. Intimate Examinations and Images (94% agreement)
We do not undertake intimate examinations remotely. We follow legal and regulatory advice which limits exchange of certain kinds of intimate images.
23. Information Continuity and Action Points (100% agreement)
During or after a remote consultation, we ensure that notes, images and other data are entered on the patient's record and appropriately coded, and that agreed next steps (e.g., investigations, referral, follow-up) are actioned.
**SECTION D: LEARNING AND IMPROVEMENT**
24. Staff Training and Development (94% agreement)
We provide training, guidance and feedback to support staff on which type of appointment is appropriate in what circumstances, and for clinical trainees on remote consulting and triage.
25. Patient Training in Digital Skills (80% agreement)
We provide support or signposting (e.g., to community provision) for patients who wish to increase their digital skills and confidence with a view to consulting remotely.
26. Evaluation and Quality Improvement (87% agreement)
We measure our performance in remote consulting services, including capturing the patient experience, and set goals for improvement.

*Showing % agreement with each item in the final round*.

## Discussion

### Summary of Main Findings

We have used selected elements of a large empirical dataset, drawn from multiple UK-based empirical studies, of the introduction and scale-up of remote consultation services both before and during the pandemic to develop the Planning and Evaluating Remote Consultation Services framework. PERCS, developed as an explanatory framework for analyzing research findings, has 7 domains—the reason for consulting, the patient, the clinical relationship, the home and family, technologies, staff, the healthcare organization, and the wider system—and considers how these domains evolve over time; it also focuses on the organization's digital maturity and digital inclusion efforts.

The PERCS framework enabled us to identify and explore how dynamic interactions between individual-, organizational-, and system-level factors influenced how remote consultation services were established and delivered (or not) in different local and regional settings. It also allowed us to surface a key paradox of the pandemic, namely the mismatch between policy vision and practical reality. Both during and beyond the pandemic, policymakers envisaged an efficient, safe, and accessible remote consultation service delivered through state-of-the art digital technologies and implemented via rational allocation criteria and quality standards. In contrast, our empirical data revealed that strategic decisions about setting up remote consultation services, allocation decisions for appointment type (phone, video, e-, face-to-face), and clinical decisions when consulting remotely were fraught with contradictions and tensions, leading to both large- and small-scale ethical dilemmas that were either unique to the remote modality or greatly exacerbated by it. These dilemmas—which included how far to use technological triage to manage demand in an under-funded, under-staffed system; when to welcome support from the patient's relatives and friends in a remote consultation; when to accept a compromised physical examination rather than bring the patient in for a full in-per son assessment; how far informational continuity might compensate for relational continuity; and how much time and resource to put into mitigating digital inequalities—could not be resolved by standard operating procedures or algorithms. Rather, dilemmas had to be managed emergently by attending to here-and-now practicalities. To complement the PERCS framework, we used a Delphi process to construct a set of principles for informing the practical ethics of its application.

Our data affirm previous research (described below) which showed that the challenges of establishing and running remote consultation services operate at multiple levels: political (various interest groups may gain or lose from the introduction of new service models), economic (costs and benefits may be unevenly distributed across the system), organizational (remote consulting requires new roles and workflows), technical (dependable links and high-quality audio and images are needed), relationally (because of altered interpersonal interactions), and clinical (patients are unique; some examinations require contact; and clinicians have deeply-held habits, dispositions, and norms).

### Strengths and Limitations

Our work to develop a multi-level theoretical framework for remote consultation services has three main strengths. First, we built on previous frameworks for organizational and technological innovation which had focused on the dynamic interaction between multiple influences in a complex system (17, 62); these have been widely-used and stood the test of time. Second, the combined empirical dataset which we used to refine and extend those frameworks was large and diverse; it included several pre-pandemic studies as well as a government-funded evaluation and two in-pandemic research studies, incorporating extensive primary data from surveys, ethnographic observations, interviews, focus groups, cross-sectoral workshops, and a Delphi panel. Third, PERCS was extensively revised as we worked through analysis of the different datasets until all elements of the data could be explained with reference to the framework.

There are, however, several limitations to this work. All our empirical studies were conducted in the UK, so the PERCS framework and linked guiding principles may not be transferable to contexts very different from the UK without further adaptation (we invite collaborations from researchers in contrasting settings). Pandemic restrictions made ethnography impossible for the later months of the study, and whilst we obtained research ethics approval to video and audiotape clinical consultations, this aspect of the work has so far proved impossible in practice. Hence our current insights are based more on what people said was happening than on direct observation. The Delphi exercise to develop guiding principles for applying the PERCS framework was undertaken on a relatively small sample and should be replicated and also tested prospectively.

PERCS is an explanatory framework, not a deterministic or predictive tool. In other words, the framework and guiding principles help prompt the development of rich narratives and contextualized explanations, including ideas about what could or might happen in the future. They are not intended to determine fixed relationships between variables or firm predictions about what *will* happen.

### Comparison With Previous Research

Findings from our in-pandemic research on remote consultation services contrast sharply with research on such services undertaken before the pandemic which—in retrospect—was incomplete, skewed and lacking granularity. This earlier research, summarized briefly below, also failed to capture the complexity, messiness and associated ethical tensions of remote consultations across a range of settings once these modalities move from being evaluated in a tightly-controlled trial setting to contributing a major part of mainstream services.

A key driver for earlier research had been the hypothesis that remote models would increase efficiency of care. For this reason, many studies had emphasized measures of efficiency including repeat appointments, staff workload (including knock-on workload for other sectors), length of consultation, and the proportion of remote appointments that get converted to face-to-face (thereby double-handling a problem), as well as addressing technical feasibility, user satisfaction, clinical quality and safety, and operational considerations (97). Study designs had included randomized controlled trials, qualitative interviews, mathematical modeling, and detailed micro-analysis of verbal interactions and physical movements. Study participants had been carefully selected, usually excluding anyone considered high-risk.

Previous research on telephone consultations is surprisingly sparse and supports no firm conclusions, though several studies have suggested that double-handling may reduce efficiency (32, 98–102). There was very little research on e-consultations prior to the pandemic, and findings were limited (12, 103–107). One quantitative survey followed up 756 e-consultations in general practice and found that most generated either a telephone (32%) or face-to-face (38%) consultation (108). In contrast, there have been dozens of randomized controlled trials comparing video with face to face appointments in low-risk patients with stable long-term conditions (109–114). Overall, patients randomized to video did no worse clinically and were no less satisfied than those randomized to usual care, and that costs (where measured) were similar. However, almost all primary studies were underpowered and had highly-selected samples. Qualitative studies had indicated high patient and staff satisfaction but frequent technical problems (113–115).

Before the pandemic, there had been very few case studies of the introduction of remote consultation services in real-world settings. Most such studies lacked descriptive detail; both the clinical condition and the technology were often described in bland and brief ways, omitting the nuances that might have explained variations in findings across studies (116). The few detailed real-world studies in the literature suggest that efforts to implement remote consultation services even without an ongoing pandemic are logistically complex, resource-intensive and often stymied by technical and regulatory challenges (18, 26, 27, 115, 117, 118). Levels of remote consulting outside the research setting were very low pre-pandemic (12, 13, 119). Reasons for this included perceived increase in workload and stress, confidentiality concerns, technical problems, and demographics (e.g., the elderly were often cited as a group who found technology difficult).

Peer-reviewed research on the process and impact of the shift to remote consultation services during the pandemic is limited at the time of writing. Observational studies documented a sharp increase in remote consultations (telephone, e- and video, in that order) during the first wave in the UK (14, 15). Many findings from these studies aligned with our own: for example, that staff experienced the shift as organizationally and professionally challenging but considered it justified for safety reasons; they later reported feeling tired and under pressure as well as concerned about the loss of in-person care, threats to the therapeutic relationship, potential for missed diagnoses especially in deprived and vulnerable groups, and a rising backlog of unmet need. In surveys of staff and patients, most described their experience of technology-mediated care as “positive” but also questioned whether remote clinical care was as good as face-to-face (3). The most common concerns voiced by NHS staff were inadequate technological infrastructure and the fact that new technologies do not work for everyone. Other studies have described problems of increased antimicrobial prescribing (120), reduced ordering of diagnostic investigations (121) and delayed diagnosis of cancer (122, 123) attributed to the pandemic-related shift to remote consultations.

### Implications for Policy, Practice, Education, and Further Research

We anticipate that the multi-level PERCS framework will have a number of uses and benefits. First, we hope it will help researchers, as well as planners and policymakers, conceptualize the introduction and delivery of remote consultation services as complex interventions in complex systems rather than as tools or technologies with predictable impact and fixed effect sizes. Many interacting factors need to be taken into account, and the fortunes of remote consultation programs will unfold differently in different circumstances. Those who lead healthcare organizations may wish to set goals to improve their digital maturity and their efforts at digital inclusion. Second, we hope that clinicians will find the framework useful when considering how best to deliver excellent care to the populations they serve—especially when managing risk. The best kind of consultation for a particular patient at a particular time, taking account of the needs of other patients and staff, cannot be decided formulaically, but we hope that the guiding principles will help inform ethical allocation decisions and high-quality remote consulting. Third, we propose that use of the framework by those designing systems of care may not only reduce digital inequalities, but also lead to reductions in wider inequalities in care and burden of treatment. Finally, we believe the framework could prove useful in both undergraduate and postgraduate education, especially for promoting rich learning through reflection on practice.

We recommend further research into the application of the framework and the principles, especially the in-depth analysis of hard cases which raise particular ethical challenges.

## Data Availability Statement

The raw data supporting the conclusions of this article will be made available by the authors, without undue reservation.

## Ethics Statement

The studies involving human participants were reviewed and approved by NHS East Midlands Leicester Central Research Ethics Committee (REC ref 20/EM0128; IRAS ID: 283196 and subsequent amendments). The patients/participants provided their written informed consent to participate in this study.

## Author Contributions

All authors contributed to the empirical studies. TG conceptualized the framework, drafted the framework with RR and (to a lesser extent) other authors, and drafted the paper. TG led the Delphi study with support from RR and GW. RR is a case site lead for Remote by Default, led the focus groups and key cross-sector workshops, and wrote vignettes. SS leads a work package on Remote by Default, and provided theoretical expertise and led senior stakeholder interviews. RB is a case site lead for Remote by Default. CP leads a work package for Remote by Default. TG, RR, RB, LM, and SW contributed clinical, some social science and technology expertise, and other authors contributed social science and technology expertise. TG is guarantor. All authors selected and supplied empirical data which illustrated and shaped the PERCS framework. All authors from RB to GW contributed approximately equally and are listed in alphabetical order. All authors have read and approved the final manuscript.

## Conflict of Interest

The authors declare that the research was conducted in the absence of any commercial or financial relationships that could be construed as a potential conflict of interest.

## Publisher's Note

All claims expressed in this article are solely those of the authors and do not necessarily represent those of their affiliated organizations, or those of the publisher, the editors and the reviewers. Any product that may be evaluated in this article, or claim that may be made by its manufacturer, is not guaranteed or endorsed by the publisher.
